# mpwR: an R package for comparing performance of mass spectrometry-based proteomic workflows

**DOI:** 10.1093/bioinformatics/btad358

**Published:** 2023-06-02

**Authors:** Oliver Kardell, Stephan Breimann, Stefanie M Hauck

**Affiliations:** Metabolomics and Proteomics Core (MPC), Helmholtz Zentrum München, German Research Center for Environmental Health (GmbH), 80939 München, Germany; German Center for Neurodegenerative Diseases (DZNE) Munich, DZNE, 81377 München, Germany; Biomedical Center, Division of Metabolic Biochemistry, LMU Munich, 81377 München, Germany; Department of Genome Oriented Bioinformatics, Technical University Munich, Wissenschaftszentrum Weihenstephan, 85354 Freising, Germany; Metabolomics and Proteomics Core (MPC), Helmholtz Zentrum München, German Research Center for Environmental Health (GmbH), 80939 München, Germany

## Abstract

**Summary:**

mpwR is an R package for a standardized comparison of mass spectrometry (MS)-based proteomic label-free workflows recorded by data-dependent or data-independent spectral acquisition. The user-friendly design allows easy access to compare the influence of sample preparation procedures, combinations of liquid chromatography (LC)-MS setups, as well as intra- and inter-software differences on critical performance measures across an unlimited number of analyses. mpwR supports outputs of commonly used software for bottom-up proteomics, such as ProteomeDiscoverer, Spectronaut, MaxQuant, and DIA-NN.

**Availability and implementation:**

mpwR is available as an open-source R package. Release versions can be accessed on CRAN (https://CRAN.R-project.org/package=mpwR) for all major operating systems. The development version is maintained on GitHub (https://github.com/okdll/mpwR) and full documentation with examples and workflow templates is provided via the package website (https://okdll.github.io/mpwR/).

## 1 Introduction

The field of proteomics advances rapidly driven by the great potential of the underlying biological data. The promise of new insights continuously motivates to develop new sample preparation techniques, improved liquid chromatography (LC)-MS setups, as well as advanced data analysis solutions. The infinite process of improving individual parts of this proteomic equation requires researchers to constantly evaluate established workflows against recent progresses. Consequently, there is a need for a standardized analysis pipeline which empowers researchers to comprehensively monitor enhancements in a fast-paced proteomic field. While existing bottom-up proteomics R packages, such as protti (Quast *et al.* 2021), MSstats (Choi *et al.* 2014), msmsEDA (Gregori *et al.* 2021), MSnbase (Gatto *et al.* 2021), and TPP (Childs *et al.* 2021) provide an extensive analysis toolbox for specific individual biological settings, no available package enables researchers to trace proteomic advances from a broader perspective across multiple analyses.

To address this issue, we developed mpwR, a user-friendly R package for comparing label-free bottom-up proteomic workflows including data-dependent acquisition (DDA) or data-independent acquisition (DIA). mpwR offers several useful functions to track crucial performance measures, such as number of identifications, data completeness, number of missed cleavages, as well as quantitative and retention time precision. This comprehensive overview is especially beneficial to emphasize common tendencies across various experiments or large-scale comparisons. As an example, mpwR provides a valuable framework for a reproducible analysis of round robin studies. Furthermore, since there is a variety of proteomic software used in the community for analyzing DDA and DIA data and since the software itself is an ever-improving variable, mpwR is compatible with common software, such as MaxQuant (Cox and Mann 2008), Proteome Discoverer, Spectronaut (Bruderer *et al.* 2015) and DIA-NN (Demichev *et al.* 2020) and thus can monitor both, intra-software, and inter-software differences. Examining and comparing software options is imperative to exploit the data in the best possible way and to guarantee reproducible results. In addition, mpwR’s functionality presents a great overview for wet-lab orientated comparisons ranging from investigating sample preparation strategies to tweaking LC-MS settings. As a result, mpwR can be used in a versatile manner and thus promises in large-scale settings to capture general tendencies and in small-scale comparisons to highlight beneficial options in the process of workflow optimization in a fast and user-friendly fashion.

## 2 Description

### 2.1 Functionalities

**Figure 1.**
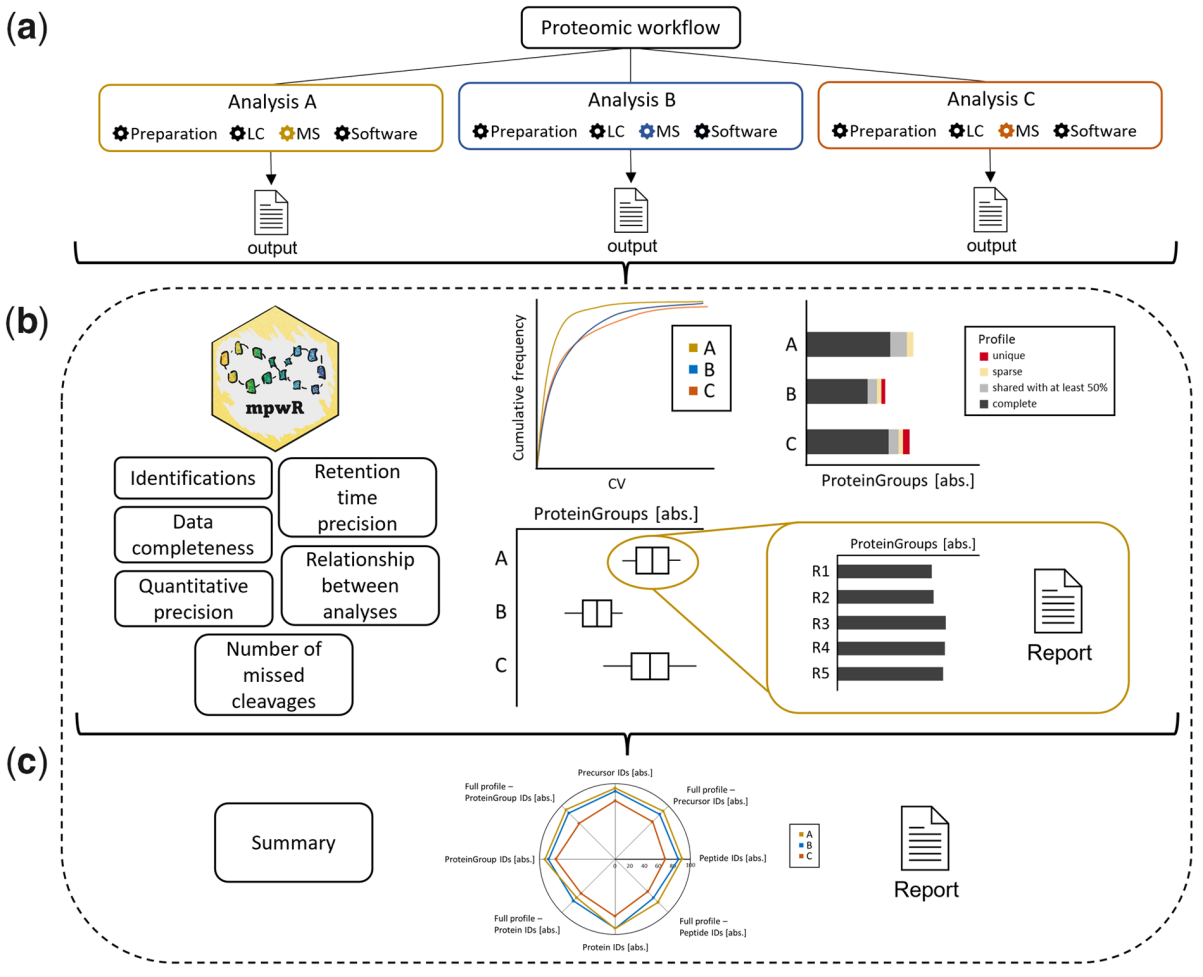
Functionalities of mpwR. An unlimited number of analyses can be submitted. In this scenario MS settings are varied in three different analyses (a). mpwR provides overview plots and details via reports and graphs for each performance characteristic (b), and summary representations for the whole comparison (c).

mpwR facilitates the comparison of proteomic workflows with a wide array of functions by analyzing important performance characteristics including number of identifications on precursor, peptide, protein and protein group level, data completeness, number of missed cleavages, as well as quantitative and retention time precision (Fig. 1).

For comparing proteomic workflows, the user can submit multiple analyses with specific adjustments to mpwR. Each analysis needs to be performed individually with two or more replicate measurements followed by a designated software analysis resulting in an output per analysis (Fig. 1a). Note, that depending on the used proteomics software one or multiple output files are required. After output submission, mpwR generates individual results per workflow analysis and performance characteristic both as report and visualization. In addition, overview graphs are available (Fig. 1b). Individual results can be accessed and modified, e.g. to remove outliers before generating the corresponding overview plots. Key figures include boxplots for comparing achieved identifications, stacked bar plots for data completeness with profile information per analysis, and cumulative density plots for highlighting differences in the coefficient of variation for retention time or quantitative data. Relationships between identifications of various analyses are visualized via upset plots (Lex *et al.* 2014). Additionally, a summary report and a radar chart can be created for a relative comparison and a comprehensive overview (Fig. 1c).

Furthermore, functions from flowTraceR, an R package which creates standardized output formats on precursor, peptide, and protein group level for MaxQuant, Proteome Discoverer, Spectronaut, and DIA-NN is incorporated in the analysis of mpwR. This combination enables researchers to perform and evaluate inter-software comparisons. The release version is available on CRAN (https://CRAN.R-project.org/package=flowTraceR) and the development version on GitHub (https://github.com/okdll/flowTraceR).

### 2.2 Applicability and implementation

mpwR supports the comparative analysis of bottom-up label-free proteomics DDA and/or DIA data from MaxQuant, Proteome Discoverer, Spectronaut, and DIA-NN. For R novices a low-level entry is provided via RMarkdown templates with thorough documentation for an mpwR data analysis pipeline for each software and for inter-software comparisons (https://okdll.github.io/mpwR/). Also, the modularized structure of mpwR allows easy workflow adjustments for a user-tailored analysis.

In addition, each core functions and visualizations are integrable into the R shiny framework for custom-made dashboards or apps. As an example, a shiny dashboard was designed for small scale comparisons (https://okdll.shinyapps.io/mpwR/). The developed dashboard allows users to quickly analyze data without any R knowledge.

Advanced R users can expand mpwRs functionalities to other proteomic software tools if the required information is accessible, e.g. precursor, peptide, and protein group information. A template for a generic input is provided. Moreover, users can add self-made modules about sequence coverage, charge state, or other characteristics of interest to the existing pipeline.

## 3 Conclusions

mpwR offers a systematic approach for comparing proteomic workflows and empowers researchers to access valuable information about identifications, data completeness, quantitative precision, and other performance indicators across an unlimited number of analyses and multiple software tools. The resulting comprehensive overview provides a sound fundament to evaluate potential proteomic advances and promises quick data analysis turnaround times in the process of workflow optimization.
